# The Roots of Problematic Polydrug Use in Emotional Problems and Suicide Behavior: A Cross-Sectional Study

**DOI:** 10.3390/brainsci15090940

**Published:** 2025-08-28

**Authors:** Tania E. Martinez, Alejandro De la Torre-Luque, Anna Pedrola-Pons, Elizabeth Suarez-Soto

**Affiliations:** 1 Department of Psychology, European University of Valencia, 46040 Valencia, Spain; esparzamartineztania@gmail.com; 2 Department of Legal Medicine, Psychiatry and Pathology, Faculty of Medicine, Office #16, 4th Floor, 3rd Pavilion, Universidad Complutense de Madrid, 2 Seneca Avenue, 28040 Madrid, Spain; 3 Department of Legal Medicine, Psychiatry and Pathology, Complutense University of Madrid, CIBERSAM ISCIII, 28040 Madrid, Spain; 4 Department of Legal Medicine, Psychiatry and Pathology, Faculty of Medicine, Complutense University of Madrid, 28040 Madrid, Spain; a.pedrola.pons@gmail.com (A.P.-P.); elizasua@ucm.es (E.S.-S.); 5 Department of Psychology, International University of Valencia, 46023 Valencia, Spain

**Keywords:** polydrug use, behavioral addictions, depression, anxiety, suicide behavior, dual diagnosis

## Abstract

**Background/Objectives**: Problematic polydrug use represents a relevant public health concern, with strong relationships with mental health problems and suicide behavior. Existing studies have just focused on problematic use, overlooking the potentially cumulative effect of coexisting substance use and addictive behaviors. This study aims to analyze the association between the polydrug use profile (no problematic use, problematic use of a single drug, and polydrug use) and mental health outcomes, specifically anxiety symptoms, depressive symptoms, and suicide behavior. **Methods**: A sample of 1307 Spanish young adults (66.2% male; *M* = 21.2 years, *SD* = 3.31) were assessed for problematic use of substances and behavioral addictions as well as for the internalizing symptoms and suicide behavior. Participants were categorized into three groups: no problematic drug use (*n* = 880), problematic single drug use (*n* = 316), and polydrug use (*n* = 111). **Results**: Results showed an increasing level in depressive symptoms and suicide behavior with polydrug use, with significant differences between groups (*p* < 0.05). Moreover, both groups of problematic use presented higher levels of anxiety than no-use participants, regardless of the number of use modalities. **Conclusions**: These findings suggest that problematic polydrug use is associated with greater clinical severity, particularly in terms of depressive symptoms and suicide behavior, while anxiety remains elevated even when a problematic single drug pattern is observed. This study highlights the importance of considering polydrug use in dual diagnosis and the need for an integrative clinical approach.

## 1. Introduction

Addictive behavior is defined as excessive, compulsive, and uncontrollable conduct that is psychologically and/or physically harmful [1]. According to this definition, addictive behaviors involve a loss of control over the use of a substance or engagement in an activity, despite experiencing significant negative consequences. These consequences extend beyond the potential physical harm commonly associated with substance use to include the profound psychological and social impact that any addictive behavior can exert, often leading to a severe deterioration in an individual’s quality of life [2]. The comorbidity between addictive behaviors and other mental health conditions has received increasing attention in recent decades due to its significant clinical and public health implications. Dual diagnosis—defined as the co-occurrence of an addictive behavior and a mental disorder [3]—has emerged as a highly prevalent and severe condition, often associated with poorer prognosis, complex treatment needs, and high healthcare costs [4,5,6].

Addictive behaviors are not only harmful in themselves, but they also frequently co-occur with psychopathology disorders such as anxiety and depression, which may elevate the risk of suicidal behavior. International data show that in 2022, 7.9% of U.S. adults experienced both a mental health and a substance use disorder [7]. Among youth, substance use—particularly alcohol, cannabis, and nicotine—has been associated with higher rates of depression, anxiety, suicidal ideation, and impulsivity traits [8,9,10].

Despite variability in epidemiological data across Europe, major depression and anxiety disorders are consistently reported as the most frequent comorbidities among substance users, with prevalence rates ranging from 12% to 80% for depression and up to 35% for anxiety disorders [3]. In the U.S., notable rates of internalizing disorders have been reported. More concretely, a nationally representative study pointed out that over 16% of individuals with lifetime history of substance use disorders developed a comorbid mood disorder or anxiety disorder [11]. Furthermore, individuals with behavioral addictions such as gambling, may show a twofold risk of developing an internalizing disorder [12].

Dual diagnosis has also been linked to suicide behavior. Data from the EDADES survey (Survey on Alcohol and Other Drugs in Spain) [13]—a biennial nationally representative survey coordinated by the Spanish Ministry of Health, which gathers nationwide data on substance use prevalence, consumption patterns, sociodemographic factors, and risk perception—indicate a stark increase in suicidal ideation, planning, and suicide attempt among individuals who consume substances. For example, among individuals aged 15–64, the prevalence of illegal drug use increases from 11.5% in the general population to 35.5% among those with suicidal ideation, 37.2% with planning, and 41.1% with attempts. Problematic alcohol use rises from 6% to 16.6%, 18.4% and 21.5%, respectively, and problematic cannabis use increases from 1.9% to 11.6%, 12.6%, and 10.9% across the same levels of suicide behavior.

Three theoretical models have been proposed to explain this comorbidity: the self-medication hypothesis [14], which suggests substance use is a strategy to relieve mental health symptoms; the substance-induced model [15], which posits that substance use triggers mental disorders through neurological changes; and the shared vulnerability model [16,17], which emphasizes common genetic, psychological, and environmental risk factors.

Beyond substance use, behavioral addictions such as gambling and gaming disorder also show strong associations with internalizing disorders. High comorbidity with anxiety and panic disorders has been reported [18], as well as increased rates of anxiety, depression, and mood disorders among individuals at risk of gambling addiction [19,20]. Problematic internet use has been associated with doubling the risk of depression [21], and excessive gaming has been linked to sadness, suicidal ideation, and suicide planning [22]. However, these studies address addictive behaviors in isolation and do not consider their interaction with polydrug use.

Notably, suicide behavior has been shown to increase with the severity of gambling addiction, and this risk is exacerbated when it coexists with problematic alcohol use [23]. Although these findings suggest a heightened vulnerability, the specific impact of polydrug use remains underexplored.

Polydrug use—defined as the concurrent or sequential use of multiple substances—is increasingly common and presents significant clinical challenges, as it exacerbates health risks, reinforces addiction mechanisms, complicates diagnosis, and hinders effective treatment [24]. According to the same report [24], 39% of the studied population aged 15–64 reported using two or more psychoactive substances in the past year while 44.3% had consumed only one and 16.8% had consumed none. Behavioral addictions (e.g., gambling-related behaviors) frequently coexist with polydrug use, observing that more than 50% of individuals with behavioral addictions show a recent use of psychoactive substances, particularly alcohol, cocaine, and cannabis [12]. These complex combinations underscore the need to examine polydrug use within the broader framework of dual diagnosis. In this regard, the impact of addictive behaviors may be evident even from earlier, clinically relevant conditions, such as those derived from persistent heavy use and problematic addictive patterns [2]. Unfortunately, the existing literature does not focus on problematic polydrug use, considering the effects of both substance-related and behavioral addictions.

In summary, dual diagnosis is associated with greater clinical severity, increased psychiatric admissions, risk behaviors, and social exclusion [3]. Its cyclical and mutually reinforcing nature makes treatment more difficult and costly [25]. While recent research has advanced our understanding of the links between addiction, internalizing disorders, and suicide behavior, little is known about the role of polydrug use in these dynamics.

The present study aims to analyze the relationship between the patterns of problematic drug use and addictive behaviors and three key clinical outcomes: anxiety symptoms, depressive symptoms, and suicide behavior. The study focuses on young adults, a population particularly vulnerable to the initiation and consolidation of addictive behaviors and the development of dual diagnosis. By examining differences across consumption profiles, the study seeks to contribute empirical evidence to inform tailored prevention strategies and early clinical interventions in this population.

Based on the previous literature, the following hypotheses were formulated: (1) Anxiety symptoms will be significantly higher in individuals with polydrug use compared to those with single use and non-use. Additionally, individuals with single use are expected to report higher anxiety levels than non-users. (2) Depressive symptoms will follow a similar pattern, with the highest scores observed in the polydrug use group, followed by the single use group, and the lowest scores in the non-use group. (3) Suicide behavior will be significantly greater among polydrug users, intermediate among single use individuals, and lowest among non-users.

## 2. Materials and Methods

### 2.1. Participants

A total of 1307 Spanish young adults aged 18 to 34 participated in this cross-sectional and observational study.

Inclusion criteria included being within the specified age range, having Spanish nationality, internet access, and providing informed consent. Participants with a sensory impairment that prevents them from completing self-report questionnaires, as well as individuals without access to the necessary electronic devices to complete the assessments, were excluded from the study.

### 2.2. Instruments

A sociodemographic questionnaire was used, asking about sociodemographic aspects such as sex, age, nationality, employment status, and educational level. Sex was recorded as a binary variable (Male/Female). Age was recorded as a continuous variable in years. Employment status was recorded as a categorical variable with three options (studying, working, and studying and working) and education level was recorded as a binary variable (secondary education or less and university studies).

To assess compulsive video gaming, the adapted version of the Game Addiction Scale (GAS-7) [26] was used, with seven items that assess the seven diagnostic criteria of compulsive video gaming and present a graduated Likert-type response format with five options, using “In the last 6 months” as a time specifier. In this research, a cutoff point of 4 was used in the GAS-7 instrument, based on the polythetic format [27]. Likewise, the reliability of the scale was good for the same sample used in this study with a Cronbach’s alpha of 0.81 [28].

Nicotine dependence was assessed with the Fagerström Test for Nicotine Dependence (FTND) [29,30], composed of six items (four dichotomous, two Likert-type with four response options). A cutoff point for heavy smoking of 7 points was used, in accordance with the Spanish version of this instrument [31]. Reliability in a Spanish sample of adults over 16 years of age was α = 0.66.

The Alcohol Use Disorders Identification Test (AUDIT) [32] was used to assess risk alcohol consumption. It consists of 10 items with a Likert-type response format with five response options, except for items 9 and 10, which have three response options. The cutoff points used are 6 for women and 9 for men based on the values proposed in a validation study in the Spanish population [33]. Furthermore, reliability was good in a sample similar to the one used in this study with a Cronbach’s alpha of 0.88 [34].

Cannabis risk use was measured with the Cannabis Abuse Screening Test (CAST) [35], composed of six Likert-type items with five response options and adapted to Spanish, with a Cronbach’s alpha of 0.75 in a Spanish sample of young adults and the cutoff point placed at a value of 7 for moderate addiction [36].

An item was also included to evaluate the frequency of cocaine, amphetamines, and gambling behaviors, using a six-point Likert scale where the response options are “Never”, “Once or less times a month”, “2 to 4 times a month”, “2 to 3 times a week”, “4 or more times a week”, and “Once or several times a day”. To identify problematic use of cocaine and amphetamines, the cutoff point was established as “2 to 4 times a month”, following the criteria of the EDADES study [24], which considers problematic cocaine use as 30 or more days a year. Given that amphetamines present similar use characteristics, the same criteria were applied. In relation to gambling, the category “2 to 4 times a month” was also used, as it is close to the threshold of frequent gambling identified in previous research [37], where individuals gambling at least once a week were included as a risk group. However, it should be noted that using frequency of use alone to determine problematic use constitutes a methodological limitation, which is further addressed in the discussion section.

The presence and severity of anxious and depressive symptomatology were evaluated with the Goldberg Anxiety and Depression Scale [38], composed of 18 dichotomous items, divided into two subscales. This scale has been adapted and validated in Spanish, but the authors do not report internal reliability coefficients [39]. Instead, they evaluated the diagnostic validity by comparing the instrument with the Paris Structured Psychiatric Structured Interview (EPEP) and obtained a sensitivity of 83.1%, a specificity of 81.8%, and a positive predictive value of 95.3%. Additionally, in order to provide an internal reliability index, Cronbach’s alpha was calculated for the study sample, obtaining a value of 0.79.

Finally, the Paykel scale of suicidal ideation [40], with five dichotomous items with a time frame set in the last year, was applied to assess suicidal behaviors. The reliability of the instrument measured through ordinal alpha in a Spanish sample is 0.93 [41].

### 2.3. Procedure

The study complies with Organic Law 3/2018 and Regulation (EU) 2016/679, guaranteeing confidentiality and appropriate data processing. In addition, the research was conducted in accordance with the Declaration of Helsinki, and the protocol was approved by the Ethics Committee of the San Carlos Clinic Hospital of Madrid, Spain (protocol C.I. 20/089-E, approved on 11 March 2020).

A retrospective cross-sectional study with convenience sampling was performed. The participants gave their informed consent prior to their participation. The dissemination of the study was carried out through institutional channels of the Universidad Complutense de Madrid (email from the Vice-Rectorate for Students, official web pages, and corporate social networks such as Twitter and Facebook).

The survey was administered online through the platform https://www.allcounted.com, which guarantees data privacy in accordance with current regulations. It was available from April 2020 to April 2022. No financial incentive or compensation was offered to participants.

### 2.4. Analysis

First, the sample was described using mean and standard deviation for continuous variables, and percentages for categorical variables. Three groups were created according to the type of consumption: no problematic use, problematic single use, and problematic polydrug use. Participants were classified as problematic single consumption users when one behavior exceeded the established clinical cutoffs and as polydrug users when two or more behaviors exceeded the cutoffs. Comparison between these groups was carried out using the chi-square test for categorical variables and, in cases in which the assumption of expected frequencies greater than 5 was not met, Fisher’s exact test was applied. For continuous variables, a one-factor anova was used. To study comparisons between pairs of groups, post hoc tests were used: Tukey in cases where the assumption of variance homogeneity was fulfilled and Games-Howell when this assumption was not met. Additionally, box plots were developed to explore the distribution and differences in the levels of anxiety, depression, and suicidal behavior according to the types of consumption.

Jamovi software (version 2.4.11) [42] was used to perform the aforementioned statistical analyses.

## 3. Results

The sample comprised 1307 participants, 33.8% of them (n = 442) being women and with mean age of 21.2 years (SD = 3.31). Overall, 73.9% of the sample were university students. No statistically significant differences were observed between the types of consumption according to sex (χ^2^(2) = 0.87, *p* > 0.05) or age (F(2, 1304) = 1.43, *p* > 0.05). Most participants (n = 880; 67.3% of sample) were classified in the no-use group.

Table 1 shows the percentages of participants who surpassed the cutoff point for problematic use on the measured substances or behaviors. Cannabis was the substance with the highest proportion of problematic use (12.6%), followed by video games (11.6%), alcohol (8.6%), and pathological gambling (5.4%). To a lesser extent, problematic risk was identified in the use of tobacco (4.3%), amphetamines (0.6%), and cocaine (0.5%).

As can be seen in Table 2, 49.9% of the participants with university education belonged to the no-consumption group, 18.6% to the problematic single consumption group, and 5.4% to the polydrug use group. In contrast, among those with secondary education or less, the corresponding percentages were 17.4%, 5.6%, and 3.1%, respectively. A significant association was found between educational level and type of consumption (χ^2^(2) = 7.17, *p* < 0.05), suggesting that participants with a higher educational level tend to have a lower risk of addictive behaviors. With respect to employment status, the majority of participants in all groups were studying (48.6%, 17.1%, and 5%, respectively), with no statistically significant relationship found with type of consumption (χ^2^(4) = 5.76, *p* > 0.05).

Regarding the specific type of use, 8.8% of the participants had exclusive problematic use of video games, followed by alcohol (6.4%), cannabis (5.7%), gambling (3.1%), and amphetamines (0.2%). No cases of exclusive problematic use of tobacco or cocaine were reported. In the category of polydrug use, cannabis was again the most prevalent behavior (7%), followed by tobacco (4.3%), video games (2.8%), gambling (2.3%), alcohol (2.2%) and, to a lesser extent, cocaine and amphetamines (0.5% in both cases).

Chi-square analyses showed significant associations between type of consumption and the presence of all addictive behaviors assessed: cannabis (χ^2^(2) = 645, *p* < 0.001), tobacco (χ^2^(2) = 630, *p* < 0.001), alcohol (χ^2^(2) = 255, *p* < 0.001), video games (χ^2^(2) = 355, *p* < 0.001), gambling (χ^2^(2) = 186, *p* < 0.001), amphetamines (χ^2^(2) = 47.3, *p* < 0.001), and cocaine (χ^2^(2) = 64.9, *p* < 0.001). These results show different patterns of distribution according to the type of use.

**Figure 1:**
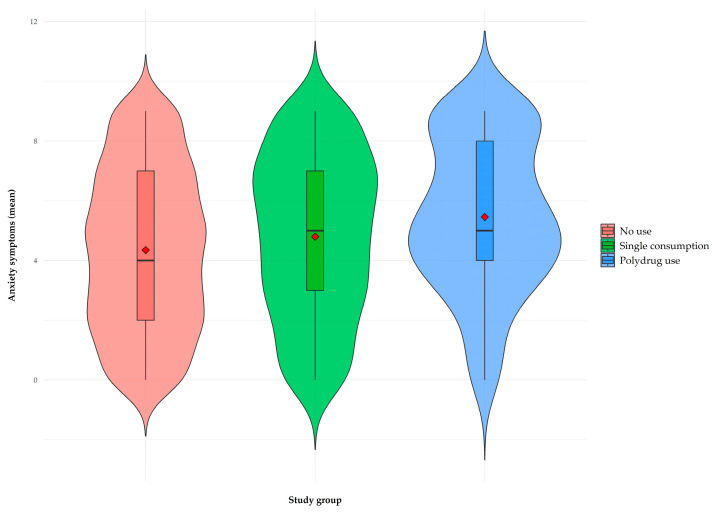
Violin plot as a function of type of consumption for anxiety symptoms. The red diamond in each box represents the average of each group; interquartile range is displayed within each violin shape.

**Figure 2:**
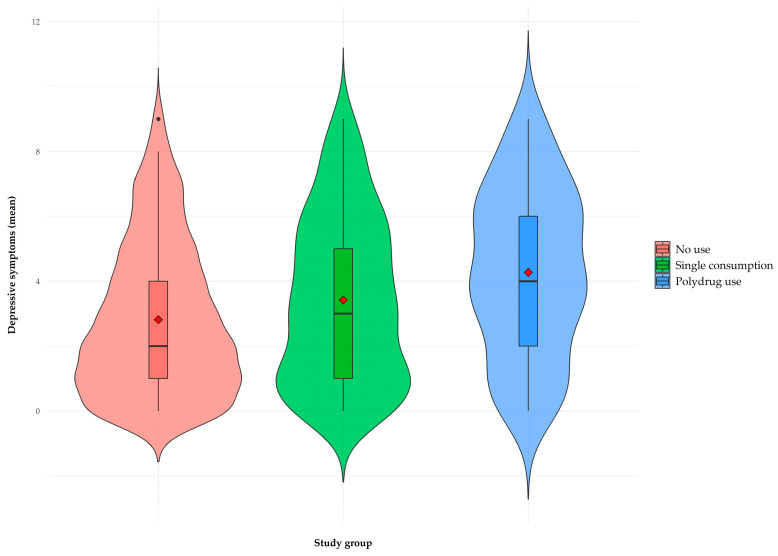
Violin plot as a function of type of consumption for depressive symptoms. The red diamond in each box represents the average of each group; interquartile range is displayed within each violin shape.

**Figure 3:**
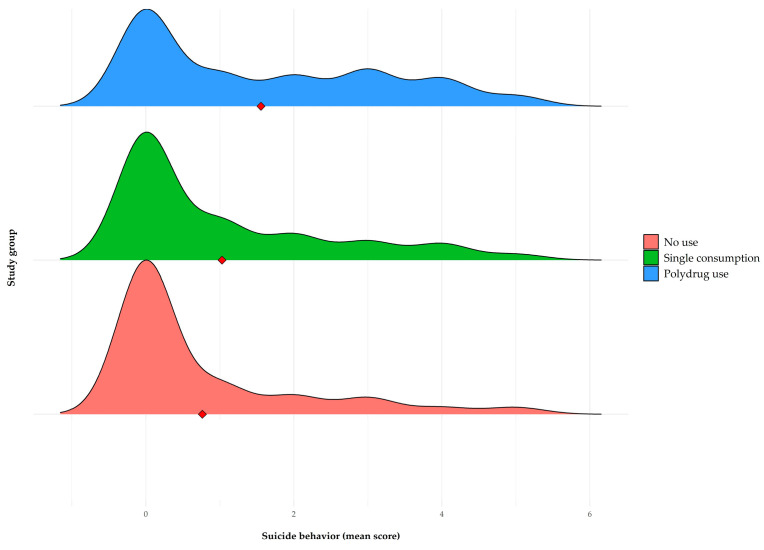
Ridgeline plot as a function of type of consumption for suicidal behavior. The red diamond in each box represents the average of each group.

In relation to internalizing symptomatology and suicidal behavior, the plots presented in Figure 1, Figure 2 and Figure 3 show an upward trend in anxiety, depression, and suicidal behavior scores as the complexity of the consumption pattern increases, from no consumption to polydrug use. These findings suggest a possible relationship between a higher degree of involvement in addictive behaviors and higher levels of psychological distress.

Finally, a one-factor analysis of variance (ANOVA) was performed to evaluate the differences between the types of consumption in the levels of anxiety, depression, and suicidal behavior. Regarding anxious symptomatology (Figure 1), significant differences were found between groups (F(2, 1264) = 9.74, *p* < 0.001, η^2^ =0,02). Post hoc comparisons (Tukey) revealed that the no-consumption group (m = 4.34, dt = 2.69) presented significantly lower scores than the problematic single consumption group (m = 4.79, dt = 2.72, *p* < 0.05) and the polydrug use group (m = 5.45, dt = 2.52, *p* < 0.001), with no significant differences between problematic single and polydrug use (*p* > 0.05).

Regarding depressive symptomatology (Figure 2), significant differences were also found between groups (F(2, 260) = 19.6, *p* < 0.001, η^2^ = 0.13). Post hoc comparisons (Games-Howell) revealed that the no-consumption group (m = 2.82, dt = 2.31) obtained significantly lower scores than the problematic single consumption (m = 3.42, dt = 2.55, *p* < 0.001) and polydrug use (m = 4.27, dt = 2.56, *p* < 0.001) groups. In addition, the polydrug use group presented significantly higher scores than the problematic single consumption group (*p* < 0.05).

Finally, in relation to suicidal behavior (Figure 3), significant differences were observed between groups (F(2, 262) = 14.5, *p* < 0.001, η^2^ = 0.1). Post hoc comparisons (Games-Howell) show that the no-consumption group (m = 0.76, dt = 1.31) obtained significantly lower scores than the single consumption group (m = 1.03, dt = 1.42, *p* < 0.05) and the polydrug use group (m = 1.56, dt = 1.63, *p* < 0.001), the latter also being significantly higher than the single consumption group (*p* < 0.01).

Taken together, these findings suggest that the accumulation of addictive behaviors is associated with greater psychological distress, especially manifested in higher levels of depression and suicidal behavior. In terms of anxiety, both the single and polydrug use groups had significantly higher scores than the no-consumption group, although no significant differences were observed between them. These results reinforce the need to consider polydrug use as a factor of higher psychological vulnerability in the young population.

## 4. Discussion

The main objective of the present study was to examine the relationship between addictive behaviors and type of consumption, comparing people with problematic polydrug use, problematic single consumption, and no problematic use, with anxious and depressive symptomatology and suicide behavior.

Regarding the percentage of cases showing addictive behaviors, the results of the study indicate that cannabis (12.6%) and video games (11.6%) present the highest levels of problematic use, followed by alcohol (8.6%) and gambling (5.4%). These figures suggest that the most common addictive behaviors in the sample correspond mainly to legal and easily accessible substances, as well as to the problematic use of video games. However, this pattern differs from the national data where in a similar age range (15 to 34 years), alcohol is the most commonly consumed substance in Spain (63.5%), followed by tobacco (33.5%) and cannabis (15.6%) [24]. These differences can be attributed to the methodological approach, as the present study assesses problematic use, whereas the national statistics collect nationally representative data, using random sampling procedures.

On the other hand, the results of this study provide relevant evidence on the relationship between problematic drug use and anxious and depressive symptomatology and suicide behavior. Regarding depressive symptomatology and suicidal behavior, the results fully confirm the hypotheses put forward. Polydrug use was significantly associated with greater depressive symptomatology and suicide behavior compared to problematic single consumption, and both groups significantly outperformed the no-consumption group. These results are in line with those consistently found in the literature, observing higher symptom severity with heavier polydrug use, as well as higher suicidal behavior [23,43,44]. Several studies have shown that young people with polydrug use often show increasing levels of risk factors for depression and suicide behavior, such as impulsivity, perceived loneliness, and lack of adaptive coping strategies [8,20,28,45].

In relation to anxiety, the results showed significant differences between the groups, although only partially. Both the single use group and the polydrug use group presented significantly higher levels of anxiety compared to the group without addictive behaviors, but no significant differences were found between them. This suggests that the presence of a single addictive behavior would be associated with an elevated level of symptomatology, without the polydrug use being a significant additional increase.

A possible explanation for this result could be due to the self-medication hypothesis [14] that postulates substance use as a coping strategy to alleviate anxiety symptoms given the anxiolytic effects of many substances such as alcohol, cannabis, and tobacco [45,46,47], which are widely present in cases of polydrug use. This would explain that anxiety levels remain in a similar range, regardless of the number of substances or behaviors involved.

These findings are consistent with previous studies that have pointed out the clinical severity in cases of dual diagnosis [4,5]. Several investigations have documented the prevalence of depressive disorders, anxiety disorders, and suicidal behavior in people with addictive behaviors [11,13,18,19,20,21,22], although without exploring differences between single and polydrug use, nor incorporating behavioral addictions. An increased risk of suicidal ideation in the combination of gambling addiction and alcohol consumption has only been reported in one study [23]. Nevertheless, this research, with an extensive sample, extends the existing evidence by pointing out two key findings. On the one hand, the definition of polydrug use relies on a wide variety of both substance-related problems and addictive behaviors. On the other hand, our study shows that not all the mental health problem dimensions analyzed are affected to the same extent by the number of addictive behaviors.

The present study has limitations that should be considered when interpreting its results. First, although validated questionnaires and psychometric tests have been used, they have not been complemented with objective measures such as biological analyses (blood, urine or hair) or activity records (economic or digital) to confirm substance use or the frequency of gambling and compulsive video gaming.

Another limitation is the cross-sectional design of the study, which prevents the establishment of causal relationships between the variables analyzed. Although significant associations between the type of consumption and symptoms of anxiety, depression and suicide behavior have been identified, it cannot be determined whether addictive behaviors are a cause or a consequence of the symptomatology. Additionally, our results should be placed within the COVID-19 pandemic context, a period during which emotional symptoms and suicidal behavior showed an increasing trend with several fluctuations, according to the virus control phases (e.g., lockdown, restriction easing) [48,49]. Even so, some studies stress enduring effects in the post-pandemic times in terms of mental health symptoms [50,51]. Anyway, longitudinal studies that analyze the evolution of consumption profile and emotional problems over time are required.

In addition, for three of the addictive behaviors included in the study (cocaine use, amphetamines, and gambling), validated tests were not used to establish a criterion for problematic use, relying only on self-report of frequency. This limits diagnostic accuracy and comparability with other studies. Future research should incorporate validated psychometric instruments to ensure greater reliability and validity.

Despite its limitations, this study provides valuable information on the relationship between polydrug use and mental health symptom comorbidity in a wide sample of young adults, with relevant implications for both research and clinical practice in the field of addictions and mental health. Given that most of the aforementioned studies have tended to analyze addictions in isolation, the results of this study reinforce the idea that polydrug use could be associated with a more severe clinical picture in terms of depressive symptomatology and suicidal behavior.

Secondly, there is some evidence on the need to conduct more exhaustive clinical assessments even in community settings, to address the complexity of problematic polydrug use and its relationship with mental health conditions. It urges a move towards comprehensive management protocols that involve a better characterization of people at risk of single and polydrug use and related clinical profiles.

Furthermore, our results are focused on a sample of young adults. Young adulthood is a life period with high vulnerability to the development of internalizing mental disorders and suicide behavior. The fact that the study sample corresponds to young adults from the community reinforces the importance of designing universal, multifactor prevention strategies adopting a lifespan perspective, since early detection and treatment of addictions and associated disorders can reduce the progression to chronicity of symptoms and improve the prognosis of patients.

In terms of implications for future research, this study lays the groundwork for further investigation of the interaction between polydrug use, psychiatric comorbidity, and suicide behavior. Given that the results indicate that anxiety does not seem to increase with the number of addictive behaviors, unlike depression and suicidal behavior, it would be interesting to longitudinally examine the mechanisms that explain this difference. Longitudinal studies could therefore determine whether anxiety precedes consumption, supporting the self-medication hypothesis. It would also be relevant to explore consumption profiles and patterns in greater detail to analyze whether certain combinations of substances or behavioral addictions imply a greater risk for the mental health problems examined or possible links between certain substances, such as the common co-use of tobacco and cannabis.

## 5. Conclusions

The findings support the idea that polydrug use is associated with greater clinical severity, especially with regard to depression and suicide behavior, and favor a more complete understanding of the interaction between multiple addictive behaviors and mental health problems, integrating variables that have been addressed in a more fragmented manner in the scientific literature. In this regard, this research provides relevant evidence to improve prevention, diagnosis, and treatment strategies, highlighting the importance of considering polydrug use and dual diagnosis together and promoting an integrative approach to this clinical reality.

## Figures and Tables

**Table 1:** Distribution of individuals according to their addictive behaviors in the sample (N = 1307).

	Whole Sample N (%)	Men n (%)	Women *n* (%)	χ^2^	*p*	Cramer’s V
Cannabis	165 (12.6)	133 (15.4)	32 (7.2)	17.6	<0.001	0.12
Video games	152 (11.6)	134 (15.45)	18 (4.1)	37.1	<0.001	0.17
Alcohol	113 (8.6)	0 (0)	113 (25.6)	242	<0.001	0.43
Gambling	71 (5.4)	65 (7.5)	6 (1.4)	21.6	<0.001	0.13
Tobacco	56 (4.3)	47 (5.4)	9 (2)	8.23	<0.01	0.08
Amphetamines	8 (0.6)	6 (0.7)	2 (0.5)	0.28	>0.05	0.01
Cocaine	6 (0.5)	4 (0.5)	2 (0.5)	0.01	>0.05	0.01

Note. The table includes the total number of participants (N) who showed problematic use of this substance or behavior, according to the cutoff points described in the Measures section. Percentage of cases (%) in comparison to the whole sample are also presented. The number of cases (*n*) and percentage of cases (%) for men and women are also displayed separately. The table presents comparisons by sex for each substance, using the χ^2^-based test. The *p* value and related effect size (using the Cramer’s V statistic) are also provided.

**Table 2:** Descriptive data by type of consumption.

	No Use	Single Consumption	Polydrug Use	χ^2^	*p*	Cramer’s V
Sex (% men)	44	16.4	5.8	0.87	>0.05	0.03
Age	21.1 (3.32)	21.1 (3.16)	21.7 (3.62)	33.2	>0.05	0.11
Studies (% university studies)	49.9	18.6	5.4	7.17	<0.05	0.07
Employment status				5.76	>0.05	0.05
Study	48.6	17.1	5			
Work	8.9	2.9	1.5			
Both	10.5	3.8	1.7			
Addictive use and behavior (% yes)						
Video games	0	8.8	2.8	355	<0.001	0.52
Alcohol	0	6.4	2.2	255	<0.001	0.44
Tobacco	0	0	4.3	630	<0.001	0.69
Cannabis	0	5.7	7	645	<0.001	0.7
Gambling	0	3.1	2.3	186	<0.001	0.38
Cocaine	0	0	0.5	64.9	<0.001	0.22
Amphetamines	0	0.2	0.5	47.3	<0.001	0.19

Note. For continuous variables, means and standard deviation are presented (in parentheses), and for categorical variables, percentage of cases is presented. Comparisons by study group are presented for each sociodemographic characteristic and addictive behavior, using the χ^2^-based test. The *p* value and related effect size (using the Cramer’s V statistic) are also provided.

## Data Availability

The data presented in this study are available on request from the corresponding author due to ethical reasons.
